# The contribution of community service during the transition to adulthood to health in adulthood

**DOI:** 10.1111/jora.12922

**Published:** 2024-02-20

**Authors:** Laura Wray‐Lake, Danielle Dunn, Valerie Freund, Deborah D. Kloska

**Affiliations:** ^1^ Department of Social Welfare University of California Los Angeles California USA; ^2^ Department of Psychology University of Michigan Ann Arbor Michigan USA; ^3^ Institute for Social Research University of Michigan Ann Arbor Michigan USA

**Keywords:** civic engagement, health, longitudinal, substance use, volunteering

## Abstract

Prior studies have linked young people's community service to indicators of health, yet little research takes the long view by connecting youth's community service to health in the next decade of life. Using a lifespan developmental lens, this study examined community service over the transition to adulthood and uses change over time in community service to predict indicators of behavioral, physical, and psychological health at ages 35 and 40. Data were taken from Monitoring the Future U.S. national multi‐cohort data spanning ages 18–40 in high school cohorts from 1976 to 1995 for age 40 (*N =* 4300) and 1976 to 2000 for age 35 (*N =* 5879). Models estimated a growth curve model for community service from ages 18 to 30 and found that the slope for community service was associated with alcohol use, binge drinking, marijuana use, healthy behaviors, and life satisfaction at ages 35 and 40, with cigarette use at age 35 only, and with self‐esteem and depressive symptoms at age 40 only. Less decline in community service over the transition to adulthood was associated with lower substance use, more healthy behaviors, and higher psychological well‐being in adulthood. This study contributes evidence that community service and health are linked across the lifespan and suggests the value of examining the long‐term implications of developmental change across adolescence and the transition to adulthood.

## COMMUNITY SERVICE DURING THE TRANSITION TO ADULTHOOD AND HEALTH IN ADULTHOOD

Community service is embedded in the fabric of U.S. society as a fundamental way people help one another, participate in organizations, and improve communities (Pancer, 2015). Synonymous with volunteering, community service is a common form of civic engagement, with 30% of adults reporting formal volunteering in 2019 and 23% in 2021, and more than half reporting informal helping (AmeriCorps, 2022). Youth community service has been on the rise across cohorts for several decades (Wray‐Lake et al., 2017), and national investment in service programs like AmeriCorps and secondary school and college missions' emphasis on service underscore the value society places on youth community service (Bass, 2013; Flanagan & Levine, 2010). Scholars across disciplines have called for policies that expand civic engagement opportunities for young people as an investment in youth's health (Institute of Medicine, 2015). In some studies, youth community service has been linked to better psychological health (Ballard et al., 2019; Chan & Mak, 2020; Hart et al., 2014; Schreier et al., 2013; Wray‐Lake, DeHaan, et al., 2019), but most of this research examines community service at a single time point or over a short time frame. From a developmental perspective, understanding change in community service during youth's formative transition to adulthood and its relationship to adult health can yield novel insight into the dynamic ways in which youth behaviors connect to later functioning.

Aligned with the theme of this *Journal of Research on Adolescence* special section, this study celebrates and honors the legacy of Dr. John Schulenberg by taking the long view on adolescence in examining how pathways of community service across the transition to adulthood inform long‐term health and functioning (Schulenberg et al., 2018; Schulenberg & Maslowsky, 2015). Using a lifespan perspective, this study seeks to move beyond static links between early behavior and later functioning to identify whether and how the developmental change in community service during the transition to adulthood is related to later behavioral, physical, and psychological health.

### Theoretical links between community service and health

As far back as Aristotle, scholars have posited that civic engagement is part of a life well‐lived. Contemporary theory also argues that social contributions are integral to health (Ballard & Syme, 2016; Keyes, 2007). In this study, we view health as multidimensional; although there are numerous definitions of health and its dimensions (e.g., Huber et al., 2011; Keyes, 2007; McCartney et al., 2019; Vogel et al., 2017), we focus our inquiry on three distinct yet overlapping dimensions of health: behavioral health (healthy behaviors, operationalized as eating well and exercising and lower rates of substance use); physical health (the condition and functioning of one's body, operationalized as illness symptoms and self‐rated health); and psychological health (mental health and well‐being, operationalized as self‐esteem, life satisfaction, and depressive symptoms). Given our study's correlational design, we do not intend to offer causal evidence that community service improves any dimension of health; however, we present some theoretical explanations, which are not mutually exclusive, for how and why community service may relate to later health. These theoretical ideas inform our inquiry into links between community service during the transition to adulthood and health in adulthood.

First, community service may improve health via building social capital, that is, supportive, relational bonds between individuals and social networks (Putnam, 2000). Indeed, civic engagement (including community service) is associated with increases in social capital and social connectedness (Creaven et al., 2017; Gray & Stevenson, 2020; Luque‐Suárez et al., 2021). In turn, social capital is robustly associated with greater physical, psychological, and behavioral health in terms of lower substance use (cf. Ehsan et al., 2019; Rodgers et al., 2019). Second, community service is thought to be internally rewarding and offers young people a sense of identity (Malin et al., 2015; Piliavin & Siegl, 2015). For example, civic engagement may offer purpose and meaning in life (Flanagan & Bundick, 2011; Thoits, 2012) and develop life skills and a sense of responsibility, which in turn, are associated with higher life satisfaction and happiness and lower depression and substance use (Hurd et al., 2014; Khasanzyanova, 2017). Third, community service and helping behaviors may have direct physiological benefits, via activation of reward processing systems, stress regulation, and mental health functioning (Piliavin & Siegl, 2015; Schreier et al., 2013). The neuropeptide oxytocin is associated with affiliative prosocial behavior and may also buffer against reactivity to social stressors (e.g., Heinrichs et al., 2012), which may in turn reduce substance use, support physical health, and increase well‐being. Fourth, research on extracurricular activities makes a time‐use argument, such that structured leisure activities like community service give adolescents constructive ways to spend time, which can benefit psychological health and deter youth from substance use (Oberle et al., 2020). Testing these mechanisms is beyond this study's scope, yet these perspectives offer reasons to expect that community service relates to better health and inform our study which links community service trajectories to later health.

Importantly, links between community service and health are unlikely to be unidirectional. However, little theory has speculated about whether or why youth's health would relate to later community service. In considering adults, some have argued that happy people volunteer more, suggesting a link from psychological health to community service (Thoits & Hewitt, 2001), and others have suggested that prospective links from community service to health may be partly due to selection effects such as prior well‐being or socioeconomic status (Lawton et al., 2021; Mohan & Bulloch, 2012). Thus, it is important for longitudinal research linking community service to later health to account for the role of health in community service and potential selection effects.

### Empirical links between community service and health

A fairly robust body of evidence supports a prospective association between community service and health, with the majority of research on older adults. For example, community service predicts lower depression up to 8 years later among adults 60 or older (Kim & Pai, 2010) and is linked to lower mortality and better physical, cognitive, and psychosocial functioning among older adults (Anderson et al., 2014; Kim et al., 2020; Milbourn et al., 2018). A nationally representative longitudinal study of adults in the United Kingdom found that community service predicted better psychological health (including life satisfaction), and associations were stronger for young adults and older adults compared to those in midlife (Lawton et al., 2021). Although less research has focused on youth, evidence shows that helping behaviors relate to increases in well‐being in college students (Geng et al., 2022; Martela & Ryan, 2016; Wray‐Lake, DeHaan, et al., 2019) and noncollege attending young adults (Fenn et al., 2023), although the latter population is rarely studied. A randomized control trial of high school students found that volunteering increased cardiovascular health (Schreier et al., 2013). Studies using National Longitudinal Study of Adolescent to Adult Health data found that adolescents' and young adults' community service predicted lower depressive symptoms and lower substance use in adulthood (Ballard et al., 2019; Kim & Morgül, 2017; Wray‐Lake, Shubert, et al., 2019).

Existing research has focused mostly on community service in relation to psychological health, with fewer studies on physical or behavioral health. This research gap merits exploration, given the prevalence and public health consequences of substance use and use disorders (SAMHSA, 2021) and preventable illness and disease (Galea & Maani, 2020). Theoretically, community service during the transition to adulthood may relate to better physical and behavioral health in the same ways as it is theorized to benefit mental health—through the cultivation of positive relational bonds with a prosocial community, development of identity and preparation for adulthood, and reduction of daily stressors (e.g., Ballard & Syme, 2016; Hart et al., 2014). Yet, community service may not be positively related to all forms of health; for example, a German study found no protective effects of community service in relation to substance use, even finding positive associations between community service and certain forms of substance use (Pavlova et al., 2019). A large study of college students found inconsistent associations between volunteer hours and health: students high in volunteering reported poor sleep and feeling more overwhelmed but were less depressed and more physically active (Lederer et al., 2015). Some studies with adults have noted small or null associations between community service and psychological health (e.g., Lühr et al., 2022), suggesting more research is needed and effect sizes must be considered for practical significance.

### Applying a lifespan perspective

Most existing studies linking community service to health for youth are cross‐sectional or use community service at a single time point to predict later health. Thus, extant research lacks robust evidence of longitudinal associations between community service and health and fails to consider the role of developmental change in community service across the transition to adulthood. A key tenet of lifespan development is that early experiences shape later experiences (Rutter, 1996), and linking early to later experiences is enhanced by considering constructs over multiple time points, rather than connecting two snapshots over a time span, as development is dynamic and not static (Schulenberg et al., 2018).

Drawing on a lifespan developmental perspective, it is important to consider the nature of developmental change in community service during the transition to adulthood to understand potential associations with health. Community service shows a steady decline on average across the transition to adulthood for U.S. youth (Wray‐Lake et al., 2017). Thus, engaging in community service becomes precipitously less frequent, on average, for U.S. youth from ages 18 to the late 20s. Community service may become a lower priority as young people navigate new social roles and life experiences related to education, work, or relationships that come with transitioning to adulthood (Settersten Jr. & Ray, 2010; Wray‐Lake & Ballard, 2023). Additionally, fewer institutionalized opportunities for community service are available during the transition to adulthood compared to adolescence, meaning that young adults have less consistent access to participating (Finlay et al., 2010; Wray‐Lake & Ballard, 2023). Thus, young people may face barriers to community service during the transition to adulthood. Meaningful individual differences in their developmental change may also emerge, as adolescents differ in levels of community service due in part to varying opportunities, access, interests, and motivations (Pancer, 2015), and these variations persist or grow across the transition to adulthood (Myers et al., 2019). Youth who can sustain some engagement in community service despite forces that precipitate a decline during the transition to adulthood may also have better health later in life.

Community service in high school may also directly relate to later health. Community service among high school seniors has been on the rise since the 1990s, likely due to an increased emphasis on service learning, mandatory service policies, and college resume building (Syvertsen et al., 2011; Wray‐Lake et al., 2017; Youniss & McIntosh, 2010). Experiences in adolescence could be formative for establishing longer‐term habits and commitments, particularly given that adolescents are exploring who they are and want to be (Flanagan, 2013). Research, although mostly dated, has shown that high school community service predicts diverse forms of civic engagement (including voting, values, community engagement, and leadership) up to 20 years later (Hart et al., 2007; Kim & Morgül, 2017; Youniss & Yates, 1999), suggesting some continuity between adolescence and adulthood. In our study, community service at the end of high school is captured by the intercept in a growth curve model, allowing us to simultaneously examine community service in high school and change in community service over the transition to adulthood in relation to health in adulthood.

## CURRENT STUDY

Using Monitoring the Future (MTF) U.S. national, multi‐cohort panel data, our study offers a deeper and more nuanced picture of how community service relates to later health by using change in community service across ages 18–30 to predict six forms of behavioral health (cigarette use, alcohol use, binge drinking, marijuana use, narcotics use, healthy behaviors), two physical health indicators (health symptoms and self‐assessed physical health), and three aspects of psychological health (depressive symptoms, self‐esteem, and life satisfaction) at ages 35 and 40. Examining health at ages 35 and 40 provides a more robust test of our hypothesis that young people who show less decline in community service over the transition to adulthood would also be more likely to have better health in adulthood. Although not our main focus, we also examined whether age 18 community service, when youth are high school seniors, was associated with adult health. Models included various covariates that could represent selection effects and offer alternative explanations for associations between changes in community service and adult health. Furthermore, to consider the prospective role of health for later community service, models included behavioral and psychological health at age 18 in relation to community service during the transition to adulthood.

## METHOD

Monitoring the Future is an ongoing national U.S. study of the epidemiology and etiology of substance use among adolescents and adults. Each year since 1976, nationally representative samples of about 16,000 12th graders have been drawn from about 135 public and private schools; approximately 2450 respondents are randomly selected for follow‐up and illicit substance users at baseline are oversampled (Miech et al., 2021; Schulenberg et al., 2021). A random half of each cohort was surveyed 1 year after high school and the other half was surveyed 2 years after high school; each half was followed biennially until age 30. Participants also completed surveys at ages 35 and 40. The study complies with standards for ethical human subjects research. A University of Michigan Institutional Review Board (IRB) approved the original study, and this secondary analysis received expedited approval from the University of California, Los Angeles (IRB #16–001324).

We utilized two subsamples: one for age 35 and one for age 40. Participants were from the nationally representative high school senior cohorts of 1976–2000 (age 35) and 1976–1995 (age 40). Age 35 data were collected between 1993–2017; age 40 data were collected between 1998–2017. On average, respondents were 18 years old at Wave 1, 19–20 at Wave 2, 21–22 at Wave 3, 23–24 at Wave 4, 25–26 at Wave 5, 27–28 at Wave 6, 29–30 at Wave 7, 35 at Wave 8, and 40 at Wave 9. Wave 1–7 measures came from one of six randomly assigned questionnaire forms at 12th grade that contained the community service item. Panel analysis weights were included to account for panel sample selection and attrition at ages 35 or 40, dependent on the age of outcomes (Patrick et al., 2022). The use of panel analysis weights requires data to be present at the final wave, and thus sample sizes were different at age 35 (*N* = 5879) and age 40 (*N* = 4300). Average retention rate across cohorts at age 35 was 49.2% (range: 35.8%–67.1%) and 48.5% at age 40 (range: 36.7%–62.2%). Sample demographics are presented in Table 1.

**TABLE 1:** Sample descriptives.

	Age 35 sample		Age 40 sample	
	Frequency	Percentage	Frequency	Percentage
Gender^a^				
Male	2526	43.0	1846	42.9
Female	3351	57.0	2452	57.0
Race^b^				
Black	397	6.8	275	6.4
Hispanic	255	4.3	162	3.8
White	4856	82.6	3608	83.9
Other race/ethnicity	319	5.4	218	5.1
Geography				
Urban	1443	24.5	1040	24.2
Rural	1805	30.7	1368	31.8
Suburban	2631	44.8	1892	44.0
College Degree by Age 30	3591	61.1	2583	60.1
Married by Age 30	3878	66.0	2949	68.6
Divorced by Age 30	675	11.5	506	11.8
Parent Education	*M* = 4.11	*SD* = 1.31	*M* = 4.06	*SD* = 1.32

*Note*: Frequencies and percentages are reported in all cases except for parent education (a continuous variable), for which mean and standard deviation are reported (“4” = some college).

^a^ Gender was only measured as a binary.

^b^ These were the only race/ethnicity categories that were measured consistently across cohorts.

### Measures

#### Age 18–30 community service

One item assessed community service in Waves 1–7: “How often do you participate in community affairs or volunteer work?” Single‐item measurement for community service or volunteering is common in the literature (Ballard et al., 2019; Kim & Pai, 2010), and this single‐item measure has been utilized in prior work (Wray‐Lake et al., 2017). The mention of participation in community affairs or volunteer work makes the item broad and potentially inclusive of formal or informal ways of volunteering and of a wide range of social causes and community organizations for which youth may engage in service. Response options were *never* (1), *a few times a year* (2), *once or twice a month* (3), *at least once a week* (4), and *almost every day* (5).

#### Age 35 and age 40 behavioral health

Measures of substance use were identical at ages 35 and 40, were dichotomized as yes/no, and included cigarette use in the past 30 days, alcohol use in the past 30 days, binge drinking (5 or more drinks in a row) in the past 2 weeks, marijuana use in the past 12 months, and use of narcotics other than heroin in the past 12 months. Healthy behaviors were assessed as an average of five items capturing frequency of eating breakfast, eating some green vegetables, eating some fruit, exercising vigorously, and getting at least 7 h of sleep; response options were *never* (1), *seldom* (2), *sometimes* (3), *most days* (4), *nearly every day* (5), and *every day* (6).

#### Age 35 and 40 physical health

Self‐rated physical health was measured by participants' rating of their physical health over the past year relative to others of their age, on a five‐point response scale: *much below average* (1), *somewhat below average* (2), *about average* (3), *somewhat above average* (4), and *much above average* (5). Five items were averaged to measure illness symptoms in the past 30 days: headache, trouble sleeping, stayed home most or all day because you weren't feeling well, saw a doctor for a physical illness or symptom, and missed work because you were sick. Response options were *none* (1), *once* (2), *twice* (3), *3–5 times* (4), *6–9 times* (5), and *10 or more times* (6).

#### Age 35 and 40 psychological health

Depressive symptoms and self‐esteem were measured on a five‐point response scale: *disagree* (1), *mostly disagree* (2), *neither* (3), *mostly agree* (4), and *agree* (5). Depressive symptoms were assessed with an average of four items: “Life often seems meaningless,” “The future often seems hopeless,” “It feels good to be alive,” and “I enjoy life as much as anyone” (*ɑ* = .82), with the latter two reverse scored. Items are similar to the Center for Epidemiologic Studies Depression Scale (CES‐D; Radloff, 1977) and have been used previously in the MTF study (e.g., Maslowsky et al., 2014). Self‐esteem was assessed by averaging four items from the Rosenberg (1965) self‐esteem scale: “I take a positive attitude toward myself,” “I feel I am a person of worth, on an equal plane with others,” “I am able to do things as well as most other people,” and “On the whole, I am satisfied with myself” (*α* = .86). Life satisfaction was a single item asking “How satisfied are you with life as a whole these days?” on a response scale from *completely dissatisfied* (1) to *completely satisfied* (7).

#### Age 18 covariates

Numerous covariates were included in models predicting community service trajectories and adult health to account for characteristics of youth who are more or less likely to select into community service and change over time (i.e., selection effects) and to account for potential alternative explanations for associations between community service and health. Age 18 sociodemographics included a binary gender variable (*male* (0) and *female* (1)); race/ethnicity (non‐Hispanic White, Black, Hispanic, and other racial/ethnic groups with non‐Hispanic White (the largest group) as reference); parental education (highest education attained by at least one parent, with response options *completed grade school or less* (1), *some high school* (2), *completed high school* (3), *some college* (4), *completed college* (5), and *graduate or professional school after college* (6)); urbanicity (rural, urban, and suburban as reference); and historical cohort (continuous variable centered at 1976). Models controlled for senior high school grade point average (GPA) (*A to B‐* (1) and *C+ to D* (0)) and religiosity (average of two standardized items: frequency of attending religious services (*never* (1), *rarely* (2), *once or twice/month* (3), and *about once/week* (4)) and importance of religion in their life (*not important* (1), *a little important* (2), *pretty important* (3), and *very important* (4)), given their links to community service and/or health (Patrick & Schulenberg, 2014; Wray‐Lake et al., 2020). We also included age 18 measures of age 35 and 40 health, where possible, in relevant models, including substance use coded as yes/no: past 30 days use of alcohol, past 30 days use of cigarettes, binge drinking in the past 2 weeks (yes/no), any past‐year marijuana use, and any past‐year narcotics use; and self‐esteem, life satisfaction, healthy behaviors, and self‐rated physical health. Depressive symptoms and illness symptoms were not measured at age 18 on the same survey form as community service. In predicting age 35 and age 40 substance use, we controlled for age 18 sensation seeking (average of two items on preference for risk‐taking, with responses *disagree* (1), *mostly disagree* (2), *neither* (3), *mostly agree* (4), and *agree* (5)), and interpersonal aggression (average of five items including hurting, threatening, and injuring others in the past 12 months, with responses *not at all* (1), *once* (2), *twice* (3), *3 or 4 times* (4), and *5 or more times* (5)), given established links to substance use (Evans‐Polce et al., 2018).

#### Young adult covariates

Youth vary substantially in their life paths across young adulthood, and key social role markers during this period may relate to community service and health. Thus, several factors were included as controls. Educational attainment was coded as whether the respondent completed an Associate's degree or higher between ages 19 and 30 (yes/no). Ever married and ever divorced were assessed with binary variables indexing whether respondents were ever married or divorced, respectively, between ages 19 and 30.

### Analytic plan

Using M*plus* 8.8 (Muthén & Muthén, 1998–2017), all models were estimated with robust standard errors (MLR) to adjust for any non‐normality, included weights to adjust for panel sample selection and attrition, and full information maximum likelihood (FIML) to handle item missingness. Model fit was assessed through root mean square error of approximation (RMSEA) of .05 or lower, and a comparative fit index (CFI) and Tucker–Lewis index (TLI) of .90 or greater (Hu & Bentler, 1999). We identified the best‐fitting latent growth curve model for community service by examining linear, quadratic, and cubic models across seven waves of data spanning ages 18–30. The intercept was estimated at age 18. The best‐fitting model was determined from Satorra–Bentler chi‐square comparisons (used with MLR estimation; Satorra & Bentler, 2010) and model fit indices described above. After identifying the best‐fitting growth curve model, we estimated four models to predict substance use at age 35 and 40, respectively, and other health indicators at age 35 and 40, respectively. Models included age 18 and young adult covariates as predictors of growth parameters. Nonsignificant correlations among covariates were removed for model parsimony, so degrees of freedom vary slightly across models. Age 35 or 40 constructs of interest were also regressed on growth parameters, controlling for covariates. Standardized path coefficients are presented, with values around .50 indicating a large effect, around .30 indicating a medium effect, and around .10 indicating a small effect (Cohen, 1992).

## RESULTS

In comparing linear, quadratic, and cubic growth curve models for community service, the quadratic model was a better fit than the linear model (Satorra–Bentler adjusted Δ*χ* ^*2*^ = 312.88, *Δdf* = 4, *p* < .001), and the cubic model did not converge. The quadratic model was selected as best‐fitting (*χ* ^*2*^ = 79.43, *df* = 19, *p* < .001, RMSEA = .023, CFI = .99, TLI = .99, SRMR = .016), aligning with previous work using MTF data (Wray‐Lake et al., 2017). The intercept for community service at age 18 was 2.07 (one or two times a month), and community service declined across the transition to adulthood, as indicated by the linear slope (*α* = −.139, *p* < .001), before leveling off in the late 20s, as indicated by the quadratic slope (*α* = .018, *p* < .001). In predictor models, the quadratic slope could not be used to predict age 35 or 40 constructs due to insufficient variance (ψ = .002, *p* < .001).

### Substance use at age 35 and 40

For substance use, the age 35 model (*χ* ^*2*^ = 989.80, *df* = 216, *p* < .001, RMSEA = .025, CFI = .933, TLI = .905, SRMR = .023) and age 40 model (*χ* ^*2*^ = 831.41, *df* = 224, *p* < .001, RMSEA = .025, CFI = .925, TLI = .897, SRMR = .024) demonstrated good fit to the data (see Tables 2 and 3). The community service slope negatively predicted age 35 past 30‐day cigarette use (*β*_age35_ = −.08, *p* < .001) and age 35 and 40 past 30‐day alcohol use (*β*_age35_ = −.08, *β*_age40_ = −.11, *p*s < .001), binge drinking (*β*_age35_ = −.07, *p* = .003, *β*_age40_ = −.068, *p* = .018), and past 12‐month marijuana use (*β*_age35_ = −.09, *β*_age40_ = −.10, *p*s < .001). Greater declines in community service across the transition to adulthood were associated with more cigarette use, alcohol use, and marijuana use at ages 35 and 40. Described in the reverse, when young people showed less decline in community service over time, they tended to have lower substance use of multiple types at ages 35 and 40. Higher community service at age 18 was associated with lower binge drinking at ages 35 and 40 (*β*_age35_ = −.06, *p* = .007, *β*_age40_ = −.06, *p* = .010) and marijuana use at age 40 (*β*_age40_ = −.07, *p* = .002). Community service was not related to later narcotics use.

**TABLE 2:** Community service growth parameters predicting age 35 substance use.

	Past 30‐Day cigarette use			Past 30‐Day alcohol use			Binge drinking			Past 12‐month marijuana use			Past 12‐month narcotics use		
	*b*	*β*	*p*	*b*	*β*	*p*	*b*	*β*	*p*	*b*	*β*	*p*	*b*	*β*	*p*
Community Service															
Intercept	0.00	0.01	0.741	0.00	0.00	0.980	−0.04	−0.06	0.007**	−0.01	−0.01	0.557	−0.01	−0.04	0.054
Slope	−0.27	−0.08	0.000***	−0.28	−0.08	0.006**	−0.25	−0.07	0.003**	−0.26	−0.09	0.000***	−0.04	−0.03	0.244
Age 18 Covariates															
Gender: Male	0.05	0.06	0.001**	0.11	0.12	0.000***	0.18	0.21	0.000***	0.07	0.10	0.000***	0.00	0.01	0.554
Cohort	−0.03	−0.05	0.005**	0.04	0.06	0.002**	0.05	0.09	0.000***	0.02	0.03	0.066	0.03	0.13	0.000***
High school GPA	−0.02	−0.02	0.359	0.04	0.04	0.030*	0.01	0.01	0.569	0.01	0.01	0.665	0.01	0.01	0.511
Religiosity	−0.02	−0.04	0.012*	−0.02	−0.03	0.104	0.00	0.00	0.875	−0.03	−0.07	0.000***	0.00	0.02	0.290
Parent education	−0.02	−0.05	0.004**	0.01	0.04	0.036*	−0.00	−0.00	0.831	0.01	0.02	0.330	0.00	0.01	0.776
Rural^a^	−0.00	−0.00	0.828	−0.09	−0.08	0.000***	−0.00	−0.00	0.813	−0.02	−0.02	0.172	−0.01	−0.02	0.371
Urban^a^	−0.01	−0.00	0.751	0.04	0.03	0.044*	−0.00	−0.00	0.926	0.01	0.01	0.514	−0.01	−0.02	0.142
Black^b^	0.04	0.03	0.134	−0.04	−0.03	0.208	0.02	0.01	0.500	0.05	0.04	0.044*	−0.03	−0.04	0.003**
Hispanic^b^	−0.08	−0.05	0.002**	−0.01	−0.01	0.793	−0.02	−0.01	0.567	0.00	0.00	0.992	−0.00	−0.00	0.965
Other race/ethnicity^b^	−0.01	−0.00	0.818	−0.04	−0.02	0.186	0.02	0.01	0.605	0.00	0.00	0.891	−0.03	−0.04	0.000***
Sensation seeking	0.01	0.03	0.062	0.01	0.02	0.257	0.00	0.00	0.964	0.02	0.02	0.001**	0.01	0.04	0.017*
Aggression	0.01	0.01	0.596	−0.02	−0.02	0.257	0.01	0.01	0.539	−0.01	−0.01	0.723	0.02	0.03	0.19
Cigarette use (30d)	0.33	0.38	0.000***	−0.03	−0.03	0.098	0.06	0.06	0.002**	0.05	0.06	0.002**	0.02	0.05	0.011*
Alcohol use (30 days)	−0.01	−0.02	0.423	0.20	0.20	0.000***	0.08	0.09	0.000***	0.01	0.02	0.333	0.01	0.02	0.236
Binge drinking	−0.01	−0.01	0.788	0.02	0.02	0.296	0.09	0.10	0.000***	0.01	0.02	0.450	−0.02	−0.05	0.028*
Marijuana use (12 months)	0.08	0.10	0.000***	0.07	0.07	0.000***	0.09	0.11	0.000***	0.17	0.22	0.000***	0.02	0.06	0.010*
Narcotics use (12 months)	0.00	0.00	0.991	0.00	0.00	0.923	0.00	0.00	0.981	0.10	0.06	0.001**	0.05	0.06	0.010*
Young Adult Covariates															
Degree by 30	−0.07	−0.09	0.000***	0.05	0.05	0.003**	−0.01	−0.01	0.699	−0.02	−0.03	0.095	−0.02	−0.05	0.009**
Married by 30	−0.09	−0.10	0.000***	−0.06	−0.06	0.000***	−0.05	−0.06	0.000***	−0.10	−0.13	0.000***	−0.03	−0.07	0.000***
Divorced by 30	0.06	0.04	0.006**	0.00	0.00	0.928	0.03	0.02	0.121	0.02	0.02	0.232	0.02	0.04	0.044

*Note*: *χ2* = 989.80, *df* = 216, *p* < .001, RMSEA = .025, CFI = .933, TLI = .905, SRMR = .023. Unstandardized (*b*) and standardized (*β*) coefficients are reported. Unstandardized *p*‐values reported. *p < .05; **p < .01; ***p < .001.

^a^ Reference group is suburban.

^b^ Reference group is White.

**TABLE 3:** Community service growth parameters predicting age 40 substance use.

	Past 30‐Day cigarette use			Past 30‐Day alcohol use			Binge drinking			Past 12‐month marijuana use			Past 12‐month narcotics use		
	*b*	*β*	*p*	*b*	*β*	*p*	*b*	*β*	*p*	*b*	*β*	*p*	*b*	*β*	*p*
Community Service															
Intercept	0.00	−0.00	0.980	−0.01	−0.02	0.570	−0.04	−0.06	0.010**	−0.04	−0.07	0.002**	−0.01	−0.03	0.167
Slope	−0.80	−0.03	0.390	−0.43	−0.11	0.001**	−0.23	−0.07	0.018*	−0.29	−0.10	0.000***	−0.07	−0.05	0.074
Age 18 Covariates															
Gender: Male	−0.01	−0.01	0.731	0.09	0.10	0.000***	0.16	0.19	0.000***	0.05	0.08	0.000***	0.01	0.023	0.270
Cohort	−0.04	−0.07	0.001**	0.07	0.09	0.000***	0.05	0.08	0.000***	0.04	0.07	0.003**	0.03	0.10	0.000***
High school GPA	−0.06	−0.06	0.015*	0.04	0.03	0.152	−0.02	−0.02	0.319	−0.02	−0.02	0.331	−0.00	−0.01	0.784
Religiosity	−0.02	−0.04	0.044*	−0.02	−0.04	0.100	0.00	0.00	0.852	−0.03	−0.07	0.001**	−0.00	−0.00	0.864
Parent education	−0.01	−0.03	0.163	0.02	0.06	0.011*	0.00	0.01	0.687	0.01	0.04	0.046*	−0.00	−0.01	0.665
Rural^a^	0.00	0.01	0.795	−0.04	−0.04	0.064	−0.01	−0.02	0.455	0.00	0.00	0.867	−0.00	−0.01	0.810
Urban^a^	−0.00	−0.00	0.944	0.04	0.03	0.140	−0.01	−0.01	0.721	0.03	0.04	0.070	−0.01	−0.01	0.566
Black^b^	0.06	0.05	0.046*	−0.06	−0.04	0.146	−0.00	−0.00	0.919	0.05	0.05	0.075	0.01	0.01	0.643
Hispanic^b^	−0.07	−0.04	0.027*	−0.02	−0.01	0.654	0.01	0.00	0.896	0.02	0.01	0.605	0.04	0.05	0.141
Other race/ethnicity^b^	0.01	0.01	0.797	−0.08	−0.04	0.085	−0.06	−0.03	0.105	0.02	0.01	0.624	−0.00	−0.00	0.974
Sensation seeking	0.01	0.03	0.094	0.02	0.04	0.097	0.01	0.03	0.150	0.01	0.04	0.035*	0.00	0.01	0.502
Aggression	0.00	0.00	0.994	−0.02	−0.01	0.631	0.02	0.01	0.540	0.00	0.00	0.881	−0.00	−0.00	0.897
Cigarette use (30 days)	0.31	0.37	0.000***	−0.02	−0.02	0.504	0.05	0.06	0.012*	0.05	0.07	0.005**	−0.00	−0.01	0.816
Alcohol use (30 days)	−0.03	−0.04	0.100	0.17	0.17	0.000***	0.07	0.08	0.001**	0.01	0.02	0.451	0.01	0.03	0.303
Binge drinking	0.02	0.03	0.200	0.04	0.04	0.123	0.11	0.12	0.000***	0.01	0.01	0.728	0.00	0.01	0.844
Marijuana use (12 months)	0.05	0.06	0.015*	0.03	0.03	0.297	0.02	0.02	0.390	0.13	0.19	0.000***	0.03	0.08	0.002**
Narcotics use (12 months)	−0.02	−0.01	0.578	0.02	0.01	0.512	0.01	0.01	0.721	0.10	0.07	0.002**	0.07	0.09	0.013*
Young Adult Covariates															
Degree by 30	−0.07	−0.09	0.000***	0.07	0.07	0.001**	−0.03	−0.03	0.134	−0.02	−0.03	0.082	−0.01	−0.01	0.568
Married by 30	−0.06	−0.07	0.000***	−0.04	−0.04	0.029*	−0.05	−0.05	0.004**	−0.06	−0.09	0.000***	−0.01	−0.04	0.129
Divorced by 30	0.00	−0.00	0.980	−0.01	−0.02	0.570	−0.04	−0.06	0.010**	−0.04	−0.07	0.002**	−0.01	−0.03	0.167

*Note*: *χ* ^*2*^ = 643.99, *df* = 181, *p* < .001, RMSEA = .024, CFI = .952, TLI = .925, SRMR = .023. Unstandardized (*b*) and standardized (*β*) coefficients are reported. Unstandardized *p*‐values reported. *p < .05; **p < .01; ***p < .001.

^a^ Reference group is suburban.

^b^ Reference group is White.

### Behavioral, physical, and psychological health at age 35 and 40

The second health model for age 35 (*χ* ^*2*^ = 894.63, *df* = 181, *p* < .001, RSMEA = .026, CFI = .944, TLI = .911, SRMR = .024) and age 40 (*χ* ^*2*^ = 643.68, *df* = 181, *p* < .001, RMSEA = .024, CFI = .952, TLI = .925, SRMR = .023) showed good fit to the data (see Tables 4 and 5). The community service slope positively predicted age 35 and 40 healthy behavior (*β*_age35_ = .09, *p* = .001 *β*_age40_ = .11, *p* < .001) and life satisfaction (*β*_age35_ = .07, *p* = .014, *β*_age40_ = .07, *p* = .040), indicating that less decline in community service was associated with more healthy behavior and life satisfaction years later, accounting for covariates. The community service slope positively predicted self‐esteem at age 40 only (*β*_age40_ = .08, *p* = .017) and negatively predicted depressive symptoms at age 40 (*β*_age40_ = −.09, *p* = .005), demonstrating that less decline in community service over the transition to adulthood was associated with higher self‐esteem and lower depressive symptoms at age 40. Additionally, higher community service at age 18 was associated with higher life satisfaction at ages 35 and 40 (*β*_age35_ = .06, *p* = .008; *β*_age40_ = .06, *p* = .030), higher self‐esteem (*β*_age35_ = .04, *p* = .049, *β*_age40_ = .09, *p* < .001) and (unexpectedly) higher illness symptoms (*β*_age40_ = .08, *p* = .002). We further examined the association between the community service intercept and illness symptoms at age 40 without covariates and found a nonsignificant association, suggesting this association was spurious. Community service was not related to later self‐reported physical health.

**TABLE 4:** Community service growth parameters predicting age 35 behavioral, physical, and psychological health.

	Healthy behaviors			Self‐rated physical health			Illness symptoms			Depressive symptoms			Self‐esteem			Life satisfaction		
	*b*	*β*	*p*	*b*	*β*	*p*	*b*	*β*	*p*	*b*	*β*	*p*	*b*	*β*	*p*	*b*	*β*	*p*
Community Service																		
Intercept	0.02	0.02	0.406	0.04	0.03	0.265	0.05	0.04	0.097	−0.04	−0.03	0.134	0.05	0.04	0.049*	0.11	0.06	0.008**
Slope	0.61	0.09	0.001**	0.28	0.04	0.194	0.04	0.01	0.803	−0.31	−0.05	0.081	0.24	0.05	0.131	0.62	0.07	0.014*
Age 18 Covariates																		
Gender: Male	−0.34	−0.20	0.000***	0.07	0.04	0.057	−0.33	−0.20	0.000***	0.11	0.06	0.001**	0.05	0.04	0.028*	−0.15	−0.07	0.000***
Cohort	0.06	0.05	0.011*	−0.06	−0.04	0.028*	0.02	0.05	0.369	−0.04	−0.04	0.026*	−0.03	−0.03	0.129	0.08	0.06	0.004**
High school GPA	0.02	0.01	0.539	0.03	0.01	0.439	−0.05	−0.03	0.152	−0.04	−0.02	0.261	−0.01	−0.00	0.874	−0.05	−0.02	0.389
Religiosity	0.15	0.02	0.375	−.018	−0.01	0.372	0.01	0.01	0.605	−0.02	−0.02	0.181	−.003	−0.01	0.792	0.04	0.03	0.075
Parent education	0.06	0.08	0.000***	0.03	0.04	0.050	−0.02	−0.03	0.122	−0.03	−0.05	0.005**	0.02	0.04	0.023*	0.02	0.02	0.306
Rural^a^	−0.01	−0.00	0.837	−0.03	−0.01	0.416	−0.01	−0.01	0.696	−0.01	−0.00	0.850	−0.01	−0.01	0.592	0.04	0.02	0.380
Urban^a^	0.04	0.02	0.235	−0.04	−0.02	0.335	−0.02	−0.01	0.471	−0.09	−0.05	0.005**	0.07	0.04	0.015*	−0.00	−0.00	0.969
Black^b^	−0.19	−0.07	0.001**	0.08	0.03	0.215	−0.17	−0.07	0.001**	0.00	0.00	0.963	0.09	0.04	0.056	−0.42	−0.12	0.000***
Hispanic^b^	−0.01	−0.00	0.945	0.05	0.01	0.514	−0.10	−0.03	0.099	−0.14	−0.04	0.014*	0.08	0.03	0.106	0.03	0.01	0.724
Other race/ethnicity^b^	−0.00	0.00	0.981	0.10	0.03	0.157	−0.05	−0.02	0.357	0.04	0.01	0.541	0.02	0.01	0.676	−0.01	−0.00	0.953
Healthy behaviors	0.24	0.29	0.000***	0.12	0.13	0.000***	−0.05	−0.07	0.001*	−0.03	−0.04	0.048*	0.04	0.06	0.011*	0.03	0.03	0.202
Self‐esteem	0.05	0.05	0.029*	0.10	0.09	0.000***	−0.05	−0.05	0.030*	−0.14	−0.14	0.000***	0.20	0.24	0.000***	0.13	0.10	0.000***
Life satisfaction	0.01	0.01	0.559	0.02	0.02	0.202	−0.01	−0.03	0.131	−0.02	−0.03	0.096	0.02	0.05	0.012*	0.03	0.04	0.023*
Young Adult Covariates																		
Degree by 30	0.13	0.07	0.000***	0.16	0.08	0.000***	−0.06	−0.03	0.056	−0.08	−0.05	0.015*	0.05	0.04	0.030*	0.06	0.03	0.161
Married by 30	0.03	0.02	0.218	0.06	0.03	0.082	−0.12	−0.07	0.000***	−0.26	−0.15	0.000***	0.10	0.07	0.000***	0.25	0.11	0.000***
Divorced by 30	−0.14	−0.05	0.001**	−0.03	−0.01	0.514	0.18	0.07	0.000***	0.15	0.06	0.000***	−0.03	−0.02	0.379	−0.12	−0.03	0.031*

*Note*: *χ* ^*2*^ = 894.63, *df* = 181, *p* < .001, RSMEA = .026, CFI = .944, TLI = .911, SRMR = .024. Unstandardized (*b*) and standardized (*β*) coefficients are reported. Unstandardized *p*‐values reported. *p < .05; **p < .01; ***p < .001.

^a^ Reference group is suburban.

^b^ Reference group is White.

**TABLE 5:** Community service growth parameters predicting age 40 behavioral, physical, and psychological health.

	Healthy behaviors			Self‐rated physical health			Illness symptoms			Depressive symptoms			Self‐esteem			Life satisfaction		
	*b*	*β*	*p*	*b*	*β*	*p*	*b*	*β*	*p*	*b*	*β*	*p*	*b*	*β*	*p*	*b*	*β*	*p*
Community Service																		
Intercept	0.06	0.05	0.076	−0.03	−0.02	0.425	0.10	0.08	0.002**	−0.04	−0.03	0.201	0.09	0.09	0.000***	0.09	0.06	0.030*
Slope	0.74	0.11	0.000***	0.23	0.03	0.391	0.38	0.06	0.083	−0.60	−0.09	0.005**	0.41	0.08	0.017*	0.65	0.07	0.040*
Age 18 Covariates																		
Gender: Male	−0.29	−0.17	0.000***	0.03	0.01	0.508	−0.30	−0.18	0.000***	0.20	0.12	0.000***	0.02	0.01	0.521	−0.11	−0.05	0.021*
Cohort	0.10	0.07	0.001**	−0.06	−0.03	0.122	−0.03	−0.03	0.254	0.03	0.02	0.405	−0.05	−0.04	0.042*	0.01	0.001	0.745
High school GPA	0.05	0.03	0.244	0.09	0.04	0.089	0.02	0.01	0.649	−0.00	−0.00	0.977	−0.02	−0.01	0.563	−0.00	−0.00	0.968
Religiosity	0.01	0.01	0.657	−0.02	−0.02	0.366	−0.02	−0.03	0.227	−0.04	−0.04	0.054	0.01	0.01	0.622	0.06	0.05	0.040*
Parent education	0.05	0.07	0.001**	0.02	0.03	0.169	−0.01	−0.02	0.345	−0.04	−0.06	0.013*	0.02	0.04	0.091	0.03	0.03	0.204
Rural^a^	−0.03	−0.02	0.401	−0.08	−0.04	0.063	−0.05	−0.03	0.190	0.00	0.00	0.992	−0.01	−0.01	0.769	0.07	0.03	0.151
Urban^a^	0.03	0.01	0.492	−0.01	−0.01	0.815	−0.07	−0.04	0.069	−0.11	−0.06	0.004**	0.04	0.02	0.250	0.02	0.01	0.725
Black^b^	−0.21	−0.08	0.001**	0.14	0.04	0.076	−0.21	−0.09	0.001**	0.03	0.01	0.653	0.12	0.06	0.034*	−0.20	−0.06	0.029*
Hispanic^b^	0.20	0.05	0.026*	0.23	0.06	0.026*	−0.16	−0.05	0.014*	−0.08	−0.02	0.309	0.12	0.04	0.041*	0.19	0.04	0.066
Other race/ethnicity^b^	−0.09	−0.02	0.278	−0.08	−0.02	0.283	0.04	0.01	0.602	0.14	0.04	0.114	−0.10	−0.04	0.187	0.09	0.02	0.416
Healthy behaviors	0.23	0.27	0.000***	0.14	0.14	0.000***	−0.06	−0.07	0.007**	−0.04	−0.05	0.030*	0.03	0.05	0.051	0.00	0.00	0.880
Self‐esteem	0.04	0.04	0.268	0.07	0.06	0.068	−0.06	−0.06	0.065	−0.18	−0.18	0.000***	0.19	0.23	0.000***	0.15	0.12	0.000***
Life satisfaction	0.00	0.01	0.842	0.03	0.04	0.060	−0.03	−0.05	0.022*	−0.02	−0.04	0.040*	0.02	0.05	0.022*	0.07	0.09	0.000***
Young Adult Covariates																		
Degree by 30	0.20	0.11	0.000***	0.23	0.11	0.000***	−0.11	−0.06	0.001**	−0.07	−0.04	0.041*	0.05	0.04	0.075	0.13	0.06	0.006**
Married by 30	0.06	0.03	0.059	0.12	0.05	0.004**	−0.04	−0.02	0.253	−0.15	−0.09	0.000***	0.08	0.06	0.002**	0.24	0.10	0.000***
Divorced by 30	−0.05	−0.02	0.299	0.02	0.01	0.787	0.02	0.01	0.651	0.07	0.03	0.130	0.03	0.02	0.390	−0.09	−0.03	0.154

*Note*: *χ* ^*2*^ = 643.678, *df* = 181, *p* < .001, RMSEA = .024, CFI = .952, TLI = .924, SRMR = .023. Unstandardized (*b*) and standardized (*β*) coefficients are reported. Unstandardized *p*‐values reported. *p < .05; **p < .01; ***p < .001.

^a^ Reference group is suburban.

^b^ Reference group is White.

### Age 18 covariates and community service trajectories

Table 6 presents age 18 covariates as predictors of community service intercept, linear slope, and quadratic slope. These associations were not the primary focus of this study, but several behavioral health indicators were concurrently associated with community service at age 18. Past 30 days cigarette use and past year marijuana use were related to lower age 18 community service. Healthy behaviors and self‐esteem were related to higher age 18 community service. Only two factors were associated with community service growth parameters: age 18 narcotics use was related to a more positive quadratic term, suggesting a greater uptick in community service in the late 20s, and self‐esteem predicted greater declines in community service followed by greater increases in the late 20s.

**TABLE 6:** Predictors of community service growth parameters.

	Intercept			Slope			Quadratic		
	*b*	*β*	*p*	*b*	*β*	*p*	*b*	*β*	*p*
Gender: Male	−0.12	−0.10	0.000***	0.08	0.33	0.000***	−0.01	−0.68	0.000***
Cohort	0.06	0.08	0.002**	0.01	0.07	0.343	−0.003	−0.28	0.083
High school GPA	0.13	0.09	0.000***	0.04	0.12	0.089	−0.01	−0.30	0.050*
Religiosity	0.18	0.26	0.000***	−0.01	−0.09	0.169	0.00	0.14	0.348
Parent education	0.09	0.18	0.000***	−0.01	−0.09	0.212	0.00	0.01	0.939
Rural^a^	0.13	0.09	0.000***	−0.02	−0.08	0.223	0.00	0.16	0.282
Urban^a^	0.05	0.03	0.155	−0.02	−0.07	0.332	0.00	0.10	0.517
Black^b^	−0.04	−0.02	0.476	0.07	0.19	0.026*	−0.01	−0.38	0.039*
Hispanic^b^	0.04	0.01	0.574	−0.04	−0.07	0.386	0.00	0.04	0.794
Other race/ethnicity^b^	0.10	0.04	0.121	−0.04	−0.07	0.371	0.01	0.15	0.395
Cigarette use (30 days)	−0.12	−0.09	0.000***	0.04	0.14	0.056	−0.01	−0.27	0.095
Alcohol use (30 days)	0.07	0.06	0.048	−0.02	−0.06	0.471	0.00	−0.01	0.969
Binge drinking	−0.06	−0.05	0.082	−0.01	−0.03	0.737	0.00	0.21	0.269
Marijuana use (12 months)	−0.13	−0.10	0.000***	0.02	0.09	0.258	−0.00	−0.11	0.539
Narcotics use (12 months)	−0.01	−0.00	0.913	−0.06	−0.11	0.054	0.01	0.32	0.012*
Sensation seeking	0.03	0.05	0.054	0.01	0.06	0.407	−0.00	−0.20	0.210
Aggression	0.01	0.00	0.895	0.02	0.08	0.316	−0.00	−0.13	0.467
Healthy behaviors	0.05	0.08	0.002**	0.01	0.08	0.350	−0.00	−0.24	0.188
Self‐esteem	0.06	0.08	0.008**	−0.05	−0.29	0.019*	0.01	0.54	0.035*
Life satisfaction	0.02	0.04	0.105	0.00	0.02	0.781	0.00	−0.67	0.689

*Note*: Parameters come from the model for substance use at age 35 with the exception of the last three health variables reported from the age 35 health model. Unstandardized (*b*) and standardized (*β*) coefficients are reported. Unstandardized *p*‐values reported. *p < .05; **p < .01; ***p < .001.

^a^ Reference group is suburban.

^b^ Reference group is White.

## DISCUSSION

Using a large, national multi‐cohort U.S. sample, this study found that youth who showed less decline in community service during the transition to adulthood also tended to report lower substance use of multiple types and better psychological health at ages 35 and 40, with fewer associations identified for physical health. We extend the existing literature on youth community service and health by examining change in community service over seven waves, using multiple indicators of health, and showing that the developmental change in community service in one decade of life links to indicators of health in the next decade. This study informs lifespan developmental research from adolescence to adulthood and suggests implications for supporting community service during the transition to adulthood.

### The transition to adulthood matters

Findings showed that change in community service over the transition to adulthood was consistently related to later health, whereas community service at age 18 was less consistently linked. Community service during high school is more emphasized in research and in practice than community service during the transition to adulthood (Flanagan & Levine, 2010). High school seniors on average have increased their levels of community service since the 1990s (Wray‐Lake et al., 2017), and many high schools have community service requirements and emphasize service opportunities, which have contributed to the heightened levels of service. Our study suggests that community service is also important to consider *beyond* high school: across the transition to adulthood, the ways in which community service is sustained, increased, or declined appear to be linked to health later in life. Although some research has documented health benefits of college students' community service and helping behavior (e.g., Geng et al., 2022; Lederer et al., 2015; Martela & Ryan, 2016), relatively few studies are inclusive of youth who did not attend college, with the notable exceptions of Fenn et al. (2023) who cross‐sectionally surveyed noncollege attending young adults and three studies that used the same nationally representative U.S. sample (Ballard et al., 2019; Kim & Morgül, 2017; Wray‐Lake, Shubert, et al., 2019). The latter studies could only examine long‐term links between community service and health at two discrete time points. Our findings thus newly document long‐term and dynamic developmental processes linking community service to health. Results align with theorizing about the transition to adulthood as a time of major life changes where youth's trajectories may contribute meaningfully to later life functioning (Schulenberg et al., 2004).

On average, community service declined from age 18 through the late 20s, meaning that young adults in general may experience challenges in maintaining their earlier, generally higher, levels of community service, likely given competing responsibilities, priorities, and fewer accessible opportunities for community service experienced during the transition to adulthood (Settersten Jr. & Ray, 2010; Wray‐Lake & Ballard, 2023). Youth who were able to counter this general declining trend tended to report better psychological health and lower substance use years later. Thus, sustaining community service during the transition to adulthood may have meaningful personal benefits that accumulate over time, an idea to be further explored using methods that allow for causal inference. Findings were more similar than different at ages 35 and 40, suggesting consistency and longevity of these associations. From a developmental perspective, these findings suggest that continuity of community service over time may offer benefits that cascade to other domains of life. Especially because many community service opportunities are short‐term or seasonal and individuals in the United States are most likely to engage in community service sporadically (Hyde et al., 2014), our examination of community service over a long period of time (18–30) highlights the potential value in building sustainable community service opportunities over the transition to adulthood.

### Community service and health

The question of whether community service benefits health over the long term is important for theoretical and practical reasons. Our study does not address this question definitively due to lack of causal design, but we advance this line of work by demonstrating that dynamic developmental change in community service, and not just service at a static point, is related to multiple indicators of health years later. Research on the development of health remains relatively separate from research on civic engagement, despite notable conceptual and empirical efforts to integrate these developmental domains (Ballard & Syme, 2016). More broadly, integrating positive youth development and risk behavior research has been a long‐standing inquiry in which more research is still needed (Schulenberg, 2006). In examining civic engagement and health, it is important to recognize that different forms of civic engagement—such as political behaviors that are system‐supporting (e.g., voting) and system‐challenging (e.g., protesting) and formal versus informal helping behavior—may have different associations with health, and continued theorizing is needed to make sense of these complex relationships (Ballard & Syme, 2016; Maker Castro et al., 2022).

Additionally, Hirshorn and Settersten (2013) persuasively argued that research on youth civic engagement often overly assumes and problematically promotes the narrative that civic engagement is good for youth immediately and into the future without substantial evidence and called for more rigorous empirical study of these questions. Our correlational study is rigorous in several ways including the use of a large national multi‐cohort sample, the long‐term longitudinal design with continuous measurement of community service; the temporal separation of community service and later health; two later assessments of multiple forms of health; and the ability to account for age 18 health in relation to changes in community service and adult health. The age 18 predictors of community service could have suggested dynamic and reciprocal links from health to community service to complement our findings that change in community service links to health. However, social and behavioral factors at age 18 did not show strong or consistent links to developmental change in community service. Additional research could explore dynamic associations between changes in behavioral or psychological health during the transition to adulthood in relation to community service (Brown et al., 2012; Fenn et al., 2022; Lawton et al., 2021; Lühr et al., 2022). Effect sizes in our study are notably small, but it is important to consider that slope effects capture the average standard deviation change in slope at each year relative to health indicators. Given our controls, we do not think our findings can be explained simply by selection, yet unmeasured factors may be at play, such as experiences, dispositions, or contextual influences in childhood or earlier adolescence or other experiences in adolescence or young adulthood that shape both community service and health. For example, experiencing poverty and other traumatic experiences may lead to less capacity and opportunity for community service and poorer health into adulthood (Heflin et al., 2019; Scheidell et al., 2018). Community service may be one potential pathway of many, which is likely not equally viable for all, toward better health across the lifespan.

Change in community service was not related to self‐rated physical health or illness symptoms at ages 35 and 40. However, we found that less decline in community service was related to higher reports of healthy behaviors, such as eating well and exercising, at ages 35 and 40. Overall in this study, community service during the transition to adulthood was not clearly associated with later physical health. In adolescence, Schreier et al. (2013) found improved cardiovascular functioning from community service after 4 months using an experimental design, and research with older adults has linked community service to improved physical health (Milbourn et al., 2018), yet research during young adulthood has been mixed. The study of college students by Lederer et al. (2015) found that more volunteer hours were related to poorer sleep and feeling more overwhelmed, but to better physical fitness, underscoring that community service might not link in the same direction to different forms of health. During the transition to adulthood, highly engaging in community service may be physically taxing, given the need to balance service with many other competing priorities. Certainly, myriad other life experiences influence physical health, and any link between community service and health is likely indirect, operating through building social relationships or purpose, which may buffer physiological stress responses or encourage other health‐promoting activities (Ballard et al., 2021; Fenn et al., 2023). Tentatively, we suggest that trajectories of community service may be more associated with behavioral and psychological health than physical health, although more experimental research is needed and more research that tests mechanisms longitudinally.

### Limitations

This study comes with limitations beyond those already acknowledged. Although a common measure of community service, our single item may underestimate community service of certain racial/ethnic or cultural groups, who engage in informal mutual aid and support but do not view it as service in a formal sense (Wray‐Lake & Abrams, 2020), and we lack insight into the types of community service and settings in which youth were involved. Asking about the participation in both community affairs and volunteer work in the item may have led to the measurement error, which is indeterminable. Higher‐quality experiences of community service—that prompt deep reflection on the root causes of social problems and facilitate authentic interpersonal reactions and collaboration with others (van Goethem et al., 2014)—may have stronger long‐term links to health. Future research should also extend beyond individual‐level associations to consider the role of community service on community‐level health, as some types of community service can perpetuate racial or social inequalities rather than addressing them (Eliasoph, 2013).

Despite our large national sample spanning multiple decades and cohorts, our sample is not fully representative of the population due to the exclusion of youth who dropped out of high school in the sampling frame and due to panel attrition, which may underrepresent individuals with serious mental health concerns and substance use disorders, young men, and people of color, who were more likely to drop out of the study. Our use of panel analysis weights helps address potential sample biases due to differential attrition, but findings may underestimate the health correlates of community service across the transition to adulthood. Our sample was U.S. specific, and we cannot comment on the extent to which these findings generalize to other country contexts. Studies from Germany, for example, have shown null, very small, or opposite‐direction associations between community service and health indicators (e.g., Lühr et al., 2022; Pavlova et al., 2019). Yet, a large mega‐analysis of European panel data showed a small, consistent association between community service and health across ages (de Wit et al., 2022), which aligns with our findings. The United States is perhaps unique in having a smaller and more precarious social safety net compared to most European countries, which may make community service more essential for meeting people's needs at a societal level (Eliasoph, 2013), but it is unclear whether this context would result in a different individual experience of volunteering.

### Implications

Especially given that young people typically experience declines in community service during the transition to adulthood, institutions and communities could look for ways to reduce barriers to community service for young adults. Although colleges and universities emphasize civic engagement including service, institutional opportunities for civic engagement are often lacking for young people after high school (Wray‐Lake & Ballard, 2023). National service opportunities like AmeriCorps or other year‐long service opportunities offer the potential for youth to build long‐term commitments to service and pay young people for their time, yet these programs are grossly underfunded, and interest and applications often significantly outstrip available positions (Bass, 2013). Community‐based nonprofits and volunteer‐based initiatives could seek to draw in youth without prior volunteer experience, and organizations could design strategies to engage or re‐engage youth after high school. Service opportunities could be designed to match youth's interests and include social components to better map opportunities onto young adults' developmental needs.

## CONCLUSION

This study documented long‐term associations between changes in community service during the transition to adulthood and health later in life. Moving beyond static associations between community service and health, this study advances lifespan developmental research and illustrates the value of considering long‐term implications of developmental change during adolescence and the transition to adulthood. Long‐term follow‐ups of young people into midlife provide needed evidence regarding what matters during adolescence and early adulthood for shaping functioning and adjustment later (Schulenberg & Maslowsky, 2015). Our study contributes further evidence that what youth do during the transition to adulthood matters and suggests that engaging in the developmental domain of civic engagement—through community service—may increase one's likelihood of taking a path to a healthy lifestyle. As Schulenberg et al. (2004) put it, the transition to adulthood is a time when young people try to “take hold of some kind of life” and youth's “ongoing interactions with the contexts represented by the various task domains depend on, and then contribute to, levels of well‐being across the transition to adulthood” (p. 1133).

## Data Availability

The data that support the findings of this study are available from the corresponding author upon reasonable request.
